# Telepsychiatry, access, and equity: accelerating mental health care for rural and low-income youth

**DOI:** 10.3389/fpubh.2025.1698682

**Published:** 2025-11-05

**Authors:** Mehek Sharma, Vikram Sharma, Dale Peeples

**Affiliations:** ^1^Medical College of Georgia at Augusta University, Augusta, GA, United States; ^2^Brown University, Providence, RI, United States; ^3^Department of Psychiatry and Health Behavior, Medical College of Georgia at Augusta University, Augusta, GA, United States

**Keywords:** telepsychiatry, rural and underserved populations, youth mental health, health equity, community initiatives, scalable, inclusive, policy

## Abstract

For rural youth, seeking mental health care often carries high stakes: in tight-knit communities where ‘everyone knows everyone,’ privacy is limited, and delays in mental health support can allow manageable symptoms to escalate into crises. Telepsychiatry provides confidential, flexible, and timely access, enabling youth to seek help early and receive support without delay. This perspective synthesizes current evidence on telepsychiatry’s benefits and challenges and highlights opportunities for growth through hybrid care models, policy reforms pertaining to payment parity and credentialing by proxy for provider licensing, digital equity initiatives, and community-based approaches for building literacy and trust, ensuring that all youth including neurodivergent persons from rural, inner-city, and low-income communities can access effective, urgent, and private mental health care.

## Introduction

The mental health of children and adolescents in the United States from low socioeconomic status (SES) backgrounds represents a growing public health concern. Mental health and mental disorders are directly associated with social determinants of health including income level, and youth from low-SES households hold a disproportionate burden of psychiatric illness including attention-deficit hyperactivity disorder (ADHD), behavioral problems, depression, and anxiety, largely driven by adverse childhood experiences (ACEs) and economic adversity (1, 2). In a national sample of U. S. adolescents, over half (58.3%) reported exposure to at least one ACE, and the vast majority experienced multiple adversities (1, 2). Delays in timely mental health care are especially concerning, as access remains severely limited for youth in rural or underserved areas, and is further compounded by privacy concerns, stigma, and provider shortages. Symptoms from ACEs can escalate into adulthood, increasing risk for crises, maladaptive coping, and long-term emotional and behavioral difficulties (3).

A nationwide shortage of child and adolescent psychiatrists has created an access crisis, with the most severe gaps concentrated in disadvantaged, impoverished, and rural areas (4). Child psychiatrists are significantly more likely to practice in high income, metropolitan counties, resulting in gaps in care across the country. In fact, between 2007 and 2016, approximately 70 percent of U.S. counties had no practicing child psychiatrists. These disparities are even more stark when broken down by state: Massachusetts had the same number of child psychiatrists as Indiana, Oklahoma, Georgia, Mississippi, and Tennessee despite having 5 times fewer children aged 0–193. This leaves more than half of the children in the U.S. with a treatable mental health disorder unable to receive treatment from a professional (4). Even when services are available, nearly one-third of referred youth never attend their first appointment, often due to transportation issues, time constraints, or stigma (4).

In response, telepsychiatry has emerged as a critical lifeline: one of the few scalable solutions capable of reaching underserved youth where they are. By providing confidential, flexible, and timely access to mental health services, it empowers youth to seek help early and discreetly (5, 6). However, to date, studies comparing the effectiveness of telepsychiatry to in-person services for children and adolescents facing both socioeconomic and geographic barriers remain limited. This article explores the current evidence on telepsychiatry’s potential to close mental health care gaps among vulnerable youth, emphasizing the need for hybrid care models, digital equity initiatives, community-based approaches, and policy reforms that ensure all young people including neurodivergent persons, regardless of geography or income, can access urgent and effective support.

## Digital psychiatric delivery (reach, privacy, equity)

### Reach

For families in rural or underserved urban areas, the nearest child psychiatrist may be hours away, making frequent visits logistically and financially challenging. Telepsychiatry offers a cost-effective alternative to traditional in-person mental health care by removing barriers of cost, proximity, and convenience that often restrict healthcare access. Naslund et al. (7) found that 60% of telepsychiatry programs were less expensive than standard in-person care. By reducing direct travel-related costs (e.g., fuel and public transport), as well as hidden costs (e.g., time off work for caregivers and missed school for children), telepsychiatry is particularly valuable for economically disadvantaged families, who would otherwise forgo mental health services for their children due to cost or scheduling barriers. Moreover, virtual appointments can significantly decrease overhead, such as physical office space and administrative support, thereby lowering costs for healthcare systems, and allowing savings to be redirected to expand services or subsidize care for un−/under-insured patients (8). This broader access can facilitate timely intervention and potentially prevent the escalation of mental health issues that might otherwise require more intensive and expensive interventions, such as emergency room visits or inpatient hospitalization (8, 9).

### Privacy

Alongside affordability and accessibility, confidentiality and safety are crucial in psychiatric care. While maintaining confidentiality can be challenging in crowded or unstable housing situations, often the case in rural, inner-city, and low-income households, providers work with families to identify the most secure location available and may use strategies such as headphones or chat features to enhance privacy (10). Telepsychiatry platforms must comply with HIPAA, using secure, encrypted communication channels, and must also have a signed Business Associate Agreement (BAA) with the platform to ensure proper data protection, as handling, transmitting, or storing Protected Health Information (PHI) on the provider’s behalf requires the platform to maintain the privacy and security of health data (11, 12). Providers verify the identity of patients and caregivers at the beginning of each session and confirm that the environment is private and free from uninvited listeners. Using structured protocols, providers evaluate risk remotely and can coordinate with local emergency services if immediate intervention is necessary, ensuring that children and adolescents receive timely and appropriate care, even when sessions are remote (11).

### Equity

Equity in access to mental health care remains a major challenge in the United States. With an acute shortage of child and adolescent psychiatrists in rural, inner-city, and low-income communities, families may have to wait months for an appointment or travel long distances to see a specialist (4). These challenges are augmented for racial and ethnic minority youth including Blacks and Hispanics, who are disproportionately affected by poverty, have significantly lower health insurance coverage, and have higher prevalence of psychiatric disorders such as depression, anxiety, substance abuse, and eating disorders. Many also experience chronic racism and ‘compounded community trauma’, or multiple and repeated traumatic events within a community, which are linked to increased rates of post-traumatic stress disorder, depression, and externalizing behaviors (13). These structural and psychosocial stressors not only increase the incidence of mental health conditions but also create significant barriers to accessing timely, appropriate, and culturally responsive care (4, 6, 13). Telepsychiatry effectively addresses these gaps by promoting equity and reducing the stigma that often prevents marginalized youth from seeking the help they need (4, 6, 13).

### Patient and caregiver satisfaction

Patient and caregiver satisfaction is a key indicator of the success of any healthcare intervention, and numerous studies cite positive experiences with telepsychiatry among low-SES children, adolescents, and their caregivers, including the convenience of at-home care, decreased travel-related stress and expense, and lower levels of anxiety in the familiar home environment (6, 9, 10). This increased comfort has been documented to lead to greater openness and engagement during sessions, facilitating effective treatment. Hispanic families find the telehealth delivery of trauma-focused cognitive behavioral therapy effective and culturally acceptable, helping overcome barriers related to stigma and access (10).

Provider communication and rapport are maintained in virtual sessions, and youth and caregivers rate their telepsychiatry experiences highly due to ease of scheduling, direct and focused interactions with providers, and the overall quality of care (9). Satisfaction is also influenced by technological reliability and the availability of private space for sessions; as such, families who experience frequent technical difficulties or lack privacy for appointments may report lower satisfaction. Nonetheless, the overall evidence suggests that telepsychiatry is well-received by low-SES families and offers a viable alternative to traditional in-person care.

### Treatment adherence and outcomes

Treatment adherence is a major challenge for low-income youth due to transportation difficulties, competing family responsibilities, and financial constraints, resulting in fragmented care (5, 9). Telepsychiatry enables remote care delivery at the fraction of the cost of traditional services without compromising quality, translating into higher satisfaction and better engagement (9). Recent studies report that telepsychiatry interventions have higher attendance rates and are generally as effective as face-to-face treatment for common mental health disorders in low-income youth (5, 7, 14, 15). While many youth from underserved backgrounds engage well with telepsychiatry, motivational interviewing and blended care models which combine digital interventions with periodic in-person support are especially valuable for those at risk of disengagement (15). A younger age, higher levels of depression, anxiety, and irritability, greater psychiatric comorbidity, and externalizing disorders such as ADHD predispose to higher dropout rates, and thus, personalization of care through hybrid models and proactive engagement strategies is essential to sustaining telehealth participation, continuity of care, and improved clinical outcomes (16).

Though research on telepsychiatry’s efficacy for lower-income youth is limited, a 2024 study linked the transition to teletherapy during the COVID-19 pandemic with significantly fewer missed appointments and depressive symptoms in youth and emerging adults (17). Another 2024 study found no significant differences in posttreatment anxiety status between virtual and in-person therapy, supporting telepsychiatry’s non-inferiority (18). Large-scale analyses using artificial intelligence techniques have shown that telepsychiatry is as effective as in-person care for depressive disorders in youth and outperforms it for anxiety (19). However, a comparison of depression severity across low and high income adults treated via telepsychiatry showed that despite both groups showing significant improvement over time, the higher income group showed significantly greater improvement in the latter time periods, suggesting that socioeconomic status may affect outcomes and further research is needed on the role of social determinants of health in outcome disparities, particularly as they relate to children and adolescents (20).

While the literature consistently demonstrates telepsychiatry’s effectiveness for youth psychiatric care, implementation decisions must carefully weigh contextual trade-offs across cost, access, quality, and equity. Table 1 provides a comparative overview of telepsychiatry and in-person modalities across key domains, including clinical efficacy, therapeutic alliance, patient acceptability, accessibility, economic factors, and equity considerations.

**Table 1:** Comparative analysis of telepsychiatry and in-person care for children and adolescents.

Domain	Telepsychiatry	In-person psychotherapy
Clinical efficacy	Pros: Non-inferior symptom reduction for depression, anxiety, trauma, and adjustment disorders compared to face-to-face care (5, 7, 14, 15). Home-based settings can reduce anxiety and improve comfort. Cons: Limited evidence for psychotic, bipolar, personality disorders, and crisis interventions. Lack of physical assessment capability may hinder risk evaluation.	Pros: Robust evidence across a wide spectrum of psychiatric disorders (severe, comorbid, treatment-resistant). It remains the “gold standard” (43). Comprehensive observation enables nuanced assessment and real-time crisis response (44). Cons: Care delays may exacerbate conditions before treatment begins (45).
Therapeutic alliance and communication scope	Pros: Alliance strength is comparable when providers are trained in digital rapport-building and patients are comfortable with the modality (46). Cons: May hinder perception of microexpressions or nonverbal cues, which may limit empathic connection for some modalities (e.g., trauma-focused or psychodynamic therapy) (47). However, screen-sharing and interactive digital tools open new therapeutic avenues (44, 48).	Pros: Rich multimodal communication (tone, gesture, spatial context, nonverbal shifts) that help clinicians sense emotional changes, discomfort, or avoidance. Clinicians can dynamically adjust pacing and modulate the session environment to optimize responsiveness (49). Cons: The visibility of attending a mental health facility may increase stigma or self-consciousness, inhibiting openness and trust in early sessions (4, 6, 7, 44).
Patient acceptability and retention	Pros: High satisfaction among younger, tech-savvy, rural, and low-SES populations (6, 10, 46). Increased attendance and greater likelihood of no cancelations. Continuity of care during crises (e.g., pandemics, natural disasters). Flexibility allows for shorter, more frequent check-ins, improving retention in ongoing care (50). Cons: “Screen fatigue” and diminished emotional connection over time (51).	Pros: High trust and acceptability among patients who value structure or personal contact (52). Cons: Logistical (transportation, travel time, schedule conflicts) and stigma can impede acceptability and retention, especially in rural, low-income populations (4). Missed sessions more likely to result in lost contact or dropout compared to telehealth rescheduling (45).
Accessibility and convenience	Pros: Significantly expands access across geographic, mobility, and socio-environmental barriers (7, 8). Enables hybrid or stepped-care models, supporting youth who transition between home, school, and clinical environments (53). Cons: Regulatory, licensure, and reimbursement constraints (e.g., cross-state) limit reach (35, 54). Connectivity issues can disrupt sessions/impair therapeutic flow. Relies on broadband quality, device access, and digital literacy (8, 9).	Pros: Integrated crisis care, on-site support (e.g., nursing, social work), and immediate clinician response (44). Predictable, structured environments can benefit adolescents needing routine or containment. Cons: Access is tied to clinician presence, clinic hours, and geographic proximity. Requires travel, time off work/school, and coordination with parental schedules (7).
Economic considerations	Pros: Decreased patient costs (travel, time off work) and system costs (facility, overhead). Reduced administrative and infrastructure burden may allow reinvestment of savings (7, 8). Cons: Platform fees, licensure compliance, and tech maintenance (7). Inconsistent payment parity limits provider participation and reduces patient access, especially for Medicaid/CHIP populations (31).	Pros: Reimbursement models for in-person care are mature, and in complex cases, the additional costs may be justified by clinical depth, safety, and integrated services (55). Cons: High operational costs (rent, utilities, staffing salaries). Patients bear transportation and time costs (7).
Equity considerations	Pros: Telepsychiatry can bridge gaps for rural, mobility-impaired, and stigmatized populations (4, 6). Reduces stigma by allowing discreet participation from home. Cons: Those without broadband, sufficient devices, private space, or tech literacy may be excluded (8, 9, 46). May not be well-adapted for neurodiverse youth needing sensory or hands-on behavioral support (22).	Pros: Accessible to digitally marginalized. Offers confidential, structured spaces for those unable to find privacy at home. Cons: Limited availability of adolescent-specialized providers, especially in underserved areas, restricts equitable access (4). Standard business hours may conflict with school or caregiving responsibilities, disproportionately affecting youth from working-class or single-parent households (7).

## Discussion: challenges and future directions

Telepsychiatry promises to increase access for rural and underserved populations; however, its scalability and equitable implementation has been hindered by challenges pertaining to clinical suitability for neurodivergent youth, trust and awareness gaps, digital tool literacy and access issues, and regulatory policies and reimbursement barriers.

### Clinical suitability for neurodivergent youth

Neurodivergent children, including those with attention-deficit/hyperactivity disorder (ADHD), autism spectrum disorder (ASD), and sensory sensitivities, may experience unique challenges with virtual sessions, making it difficult to build rapport when distractions are present at home (6, 14). Additionally, maintaining attention and steady participation can be especially challenging for neurodivergent youth, leading to decreased efficiency and productivity (21). Psychiatrists report difficulty in reading non-verbal cues and maintaining the relational elements critical to therapy, such as empathy and support (21).

The clinical suitability of telepsychiatry for neurodivergent youth can be made possible by utilizing hybrid models that combine in-person and virtual visits to promote daily living skills, social participation, and well-being for adolescents with ASD. Furthermore, incorporating non-verbal tools (e.g., images, digital apps, written communication) during telepsychiatry sessions can build trust and understanding in autistic individuals (22). Due to the heterogeneity of neurodevelopmental disorders, it is important that providers working with neurodivergent youth be trained in adapting virtual care delivery to developmental needs and diverse family dynamics to ensure inclusivity rather than a one-size-fits-all approach.

### Telepsychiatry literacy and community trust

Low-income youth face challenges with digital literacy, reducing effective engagement with telepsychiatry (8). Community-based initiatives that provide devices and digital literacy training have shown to improve engagement and adherence among underserved and marginalized groups (23). Gaps in evidence-based guidelines to ensure trainees in health service psychology receive comprehensive instruction in telepsychiatry practices leave many providers underprepared to deliver virtual care effectively or to build the trust necessary for engaging diverse populations in treatment decisions.

Several community-based models have emerged as viable pathways for telepsychiatry expansion and in building patient trust in this care approach. Efforts aimed at establishing mandated programs for trainees in health service are crucial so that they are equipped to build trust in treatment decisions (24). The 2021 Health Resources and Services Administration (HRSA) funded a pilot program for the National Telehealth Resource Center for Technology to help four state communities (Alaska, Michigan, Texas, and West Virginia) expand digital health services, particularly in rural and underserved areas (25). Federally Qualified Health Centers (FQHCs), highlighted as trusted institutions already embedded within medically underserved communities, can continue to integrate telepsychiatry into their service delivery. These centers are significantly more likely than other providers to offer patient appointments to Medicaid enrollees and provide sliding scale options for uninsured patients, serving as critical engagement channels for families and adolescents new to virtual psychiatric care (26). Embedding telepsychiatry in community structures demonstrates institutional support, legitimacy and safety, and builds confidence among patients, increasing adoption and engagement.

### Digital access and broadband divide

It has been long established that rural and underserved youth lack reliable internet or devices due to financial constraints; as a result, they miss out on opportunities for remote healthcare presented by telehealth (8, 9). Zahnd et al. found that no rural–urban commuting group met the Healthy People 2020 (HP2020) objective to provide 83.2% of the entire US population access to Broadband access (27). Isolated rural areas had broadband access at 70%, and pronounced disparities were observed in geographically isolated areas with larger Black and American Indian/Alaska Native populations, a trend that mirrors social gradients in health (27).

Broadband access initiatives, such as the Rural Health Care Program and the Department of Agriculture’s Distance Learning and Telemedicine Program, can further help bridge the digital divide in low-income and rural areas, allowing the youth most in need access to mental health care (28). By enhancing connectivity, these programs facilitate improved access to mental health services through telepsychiatry. This, in turn, accelerates diagnosis, medication management, and integration of therapeutic interventions, thereby reducing gaps in care, alleviating behavioral challenges, and decreasing caregiver burden.

### Policy and regulatory considerations

Telepsychiatry’s growth is limited by administrative challenges such as non-uniform payment parity laws across states. Many stakeholders view payment parity - requiring equal reimbursement for telehealth and in-person care - as a motivating factor for sustaining telehealth use, with mandated parity linked to a 2.5-percentage-point increase in telemedicine utilization (29) and 124% higher odds of video-based telehealth use compared to non-parity states (30). However, many parity statutes like the Mental Health Parity and Addiction Equity Act (MHPAEA) do not fully extend to Medicaid/CHIP, resulting in inconsistent behavioral health protections for the populations with the highest pediatric mental health needs (31). Future state reforms should explicitly require state Medicaid and CHIP programs to reimburse telepsychiatry visits, including synchronous (video), asynchronous (messaging), and clinically appropriate audio-only modalities at rates equal to in-person visits whenever possible, to ensure consistent access and equitable youth telepsychiatry delivery. To operationalize these reforms, state Medicaid agencies must update their Medicaid State Plans or submit State Plan Amendments (SPAs) to the Center for Medicare & Medicaid Services (CMS) detailing expanded telepsychiatry coverage and reimbursement policies. States should incorporate billing codes that cover all telepsychiatry modalities, reflecting the technology realities faced by underserved and rural youth (32).

Recent federal policy changes introduced through the One Big Beautiful Bill Act are expected to reshape Medicaid financing and eligibility, potentially influencing how states prioritize mental health services. Section 71401 of H. R. 1 appropriates $50 billion to the CMS, to be distributed to states over 5 years through the Rural Health Transformation Program. Although the legislation makes permanent pre-deductible telehealth coverage for certain private high-deductible health plans, it does not extend comparable protections or funding assurances to Medicaid or CHIP (33). Given that rural youth especially rely on these programs for health insurance, Medicaid’s Early and Periodic Screening, Diagnostic, and Treatment (EPSDT) benefit remains vital for behavioral health coverage federally, and should be preserved to reduce disparities in telepsychiatry access (34).

Current licensing restrictions further limit telepsychiatry’s reach, with only a subset of states permitting true telehealth license reciprocity. This limitation disproportionately impacts rural areas near state borders where nearby providers and patients may reside in different states (35). States could address this by adopting a behavioral-health-specific compact modeled after the Interstate Medical Licensure Compact (IMLC), which has been associated with greater out-of-state telehealth use (36, 37). Federally, the Health Resources and Services Administration (HRSA) and Substance Abuse and Mental Health Services Administration (SAMHSA) could fund pilot programs enabling “credentialing by proxy” for pediatric telepsychiatry across state lines.

Expanding evidence-based integrated care models such as the Collaborative Care Model (CoCM) through telepsychiatry can build workforce capacity and improve early identification and intervention for mental health conditions (38). Telepsychiatry enables psychiatric consultants to engage remotely with primary care teams, overcoming geographic barriers and increasing access to specialized mental health expertise in rural and underserved areas, while preserving the team-based, measurement-driven nature of CoCM (39). All state Medicaid programs should reimburse CoCM at parity with Medicare, and North Carolina’s recent emphasis on CoCM, including capacity-building funds and practice support, offers a replicable roadmap for other states (40).

Similarly, Child Psychiatry Access Line models can further improve access in underserved areas. Supported by federal Pediatric Mental Health Care Access Program grants distributed in 49 states, these programs leverage psychiatric teleconsultation and referral systems to improve timely access to specialized pediatric psychiatric care. Although focused primarily on primary care and emergency departments, these access lines can expand to support early childhood behavioral health, and care for autism and intellectual disabilities (41).

Finally, telepsychiatry reimbursement should be tied to quality and outcome measures, including reduced hospitalizations, fewer emergency visits, and improved functional status to promote value-based care. Evidence shows that outpatient telepsychiatry was associated with 38% fewer inpatient hospitalizations and 17.9% fewer emergency visits among youth, suggesting that value-based reimbursement could reduce system-wide costs (42).

Taken together, these policy changes can greatly enhance telepsychiatry accessibility, sustainability, and equity for youth - particularly for Medicaid/CHIP beneficiaries and rural populations - while fostering integrated, high-quality mental health care services.

## Conclusion

By bridging geographic and logistical barriers, telepsychiatry offers significant promise for expanding access to and improving care for socioeconomically disadvantaged youth who often face greater barriers to traditional mental health care, including cost, limited local service availability, and additional psychosocial burdens. Despite its demonstrated effectiveness and convenience, its widespread implementation is constrained by state licensing policies, disparities in technology access, and inconsistent reimbursement structures. While current studies show promise in improving emotional and behavioral outcomes among youth, the long-term impact of telepsychiatry in these communities remains unexplored, and additional longitudinal studies are needed to assess sustained effectiveness, engagement, and integration into community-based systems of care. Consistent policy frameworks will be essential to support long-term use and equitable reach of telepsychiatry for rural and low-income populations.

## Data Availability

The original contributions presented in the study are included in the article/supplementary material, further inquiries can be directed to the corresponding author.
